# Temporal bone paragangliomas: 15 years experience^☆^

**DOI:** 10.1016/j.bjorl.2016.11.001

**Published:** 2016-12-08

**Authors:** Mehmet Düzlü, Hakan Tutar, Recep Karamert, Furkan Karaloğlu, Muammer Melih Şahin, Mehmet Göcek, Mehmet Birol Uğur, Nebil Göksu

**Affiliations:** a Gazi University Faculty of Medicine, Department of Otorhinolaryngology, Ankara, Turkey; b Ankara Occupational Diseases Hospital, ENT Clinic, Ankara, Turkey

**Keywords:** Tympanomastoid paraganglioma, Tympanojugular paraganglioma, Temporal bone paraganglioma, Paraganglioma timpanomastóideo, Paraganglioma timpanojugular, Paraganglioma do osso temporal

## Abstract

**Introduction:**

Temporal bone paragangliomas (TBPs) are benign tumors arising from neural crest cells located along the jugular bulbus and the tympanic plexus. In general surgical excision, radiotherapy and wait-and-scan protocols are the main management modalities for TBPs.

**Objective:**

In this paper we aim to present our clinical experience with TBPs and to review literature data.

**Methods:**

The patients who were operated for tympanomastoid paraganglioma (TMP) or tympanojugular paraganglioma (TJP) in our clinic in the last 15 years were enrolled in the study. A detailed patient's charts review was performed retrospectively.

**Results:**

There were 18 (52.9%) cases with TMPs and 16 (47.1%) cases with TJPs, a total of 34 patients operated for TBPs in this time period. The mean age was 50.3 ±  11.7 (range 25–71 years). The most common presenting symptoms were tinnitus and hearing loss for both TMPs and TJPs. Gross total tumor resection was achieved in 17 (94.4%) and 10 (62.5%) cases for TMPs and TJPs, respectively. Five patients (31.2%) with TJP experienced facial palsy following the operation. For all the patients the mean follow-up period was 25.8 months (range 4–108 months).

**Conclusion:**

In conclusion, based on our findings and literature review, total surgical excision alone or with preoperative embolization is the main treatment modality for TBPs. However radiotherapy, observation protocol and subtotal resection must be considered in cases of preoperative functioning cranial nerves, large tumors and advanced age.

## Resumo

**Introdução:**

Paragangliomas do osso temporal (POT) são tumores benignos derivados de células da crista neural localizadas ao longo do bulbo jugular e do plexo timpânico. Em geral, a excisão cirúrgica, a radioterapia e os protocolos de acompanhamento com estudos por imagem são as principais modalidades de conduta para o POT.

**Objetivo:**

Apresentar nossa experiência clínica com POT e revisar os dados da literatura.

**Método:**

Os pacientes que foram submetidos a cirurgia para paraganglioma timpanomastoideo (PTM) ou paraganglioma timpanojugular (PTJ) em nossa clínica nos últimos 15 anos foram incluídos no estudo. Realizou-se retrospectivamente uma revisão detalhada dos prontuários dos pacientes.

**Resultados:**

Houve 18 (52,9%) casos com PTM e 16 (47,1%) casos com PTJ, portanto, um total de 34 pacientes operados para POT nesse período. A idade média foi de 50,3 ± 11,7 (intervalo de 25-71 anos). O sinais e sintomas de apresentação mais comuns foram o zumbido e perda auditiva, tanto para PTM quanto para PTJ. A ressecção tumoral completa foi obtida em 17 (94,4%) e 10 (62,5%) casos para PTM e PTJ, respectivamente. Cinco pacientes (31,2%) com PTJ apresentaram paralisia facial decorrente da cirurgia. Para todos os pacientes o tempo médio de seguimento foi de 25,8 meses (intervalo 4-108 meses).

**Conclusão:**

Com base nos nossos dados e na revisão da literatura, a excisão cirúrgica total isolada ou com embolização pré-operatória é a principal modalidade de tratamento para POT. No entanto, a radioterapia, o protocolo de observação e a ressecção subtotal devem ser considerados no caso de nervos cranianos funcionais no pré-operatório, grandes tumores e idade avançada.

## Introduction

Paragangliomas (PGs) are benign tumors arising from neural crest cells located on major neurovascular structures.^1^, ^2^ Head and neck paragangliomas are also referred to as glomus tumors. In the order of incidence glomus caroticum (60%), glomus jugulare (23%), glomus vagale (13%) and glomus tympanicum (6%) are the most commonly seen locations in the head and neck region.^3^, ^4^ Additional less common sites of origin in the head and neck are: laryngeal, nasal, orbital and paranasal region.^3^, ^4^, ^5^ Most of the PGs in the head and neck region are non-functional, as they do not secrete catecholamine.^6^ ‘Glomus’ is actually an inaccurate term used to define paragangliomas. Therefore tympanomastoid paraganglioma (TMPs) and tympanojugular paraganglioma (TJPs) are more accurate terminology instead of glomus tympanicum and glomus jugulare, respectively.^7^ TMP and TJP are together classified as temporal bone paragangliomas (TBPs). In general the incidence of TBPs is 1/1,000,000 per year and commonly seen unilateral in sporadic pattern.^8^, ^9^ However multiple PGs including TBPs tend to be of the heredofamilial variety, which account for 10% of cases.^10^ TBPs may present with the symptoms of tinnitus, hearing loss, otorrhea, facial paralysis, dysphagia and dysphonia.^11^ In general total/subtotal surgical excision, conventional fractionated external beam or stereotactic radiotherapy (SRT) and wait-and-scan protocols are main management modalities for TBPs.^11^ In this paper we aim to present our clinical experience with TBPs that were operated in our clinic and to compare these findings with the literature data.

## Methods

### Study design

**Figure 1:**
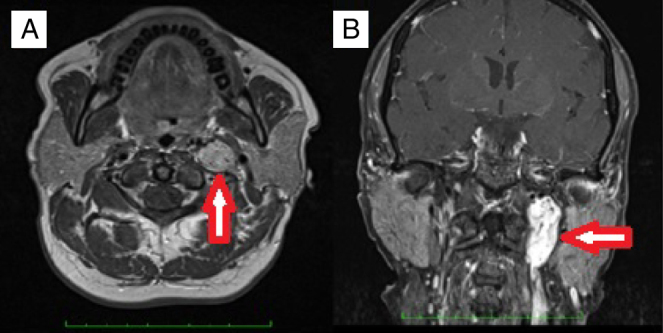
A case of left sided TJP with typical salt and pepper appearance; meaning areas of high and low intensity on MRI. (A) An axial section of T1 weighted sequences. (B) A coronal section of T1 weighted fat suppressed sequences.

**Figure 2:**
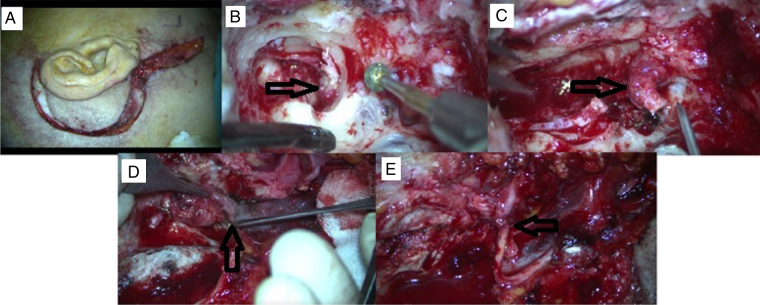
A case of TJP presented with HB grade 5 facial nerve palsy and undergone gross total tumor excision via IFTA-A approach after preoperative embolization. (A) Post-auricular incision with cervical extension. (B) Tumor extension is seen in hypotympanium during mastoidectomy. (C) Facial nerve is sacrificed which is invaded by tumor at stylomastoid foramen region. (D) Tumor originating from jugular bulbus. (E) CN 7–12 anastomosis.

This study was conducted in the Otorhinolaryngology Department of Gazi University Faculty of Medicine. Approval was granted from the University Ethical Committee (12.01.2015 Decision Number: 16). The medical records of the patients undergoing operation with the diagnosis of temporal bone paragangliomas (TMPs and TJPs) between the years 2000 and 2015 were retrospectively reviewed. The patients who were only managed with SRT or wait-and-scan protocols were not enrolled in this study. The other exclusion criterion was lacking definitive histopathological diagnosis. A through otorhinolaryngologic examination was performed. The House-Brackmann (HB) grading system was used to determine facial nerve function.^12^ A magnetic resonance imaging (MRI) was routinely performed before the operation (Fig. 1). Fisch classification^13^, ^14^ was used for staging tumors. Digital Subtraction Angiography (DSA) was performed for differential diagnosis or preoperative embolization. PVA (polyvinyl alcohol) particles were used for embolization. Blood and urine catecholamine levels were not routinely studied. Audiometric examination was performed with pure tune audiometry. All the operations were performed under general anesthesia. Erythrocyte suspension (ES) was prepared before the operation. Simple or modified radical mastoidectomy (MRM) and tympanoplasty after a post-auricular incision was performed for TMPs whereas infratemporal fossa type A (ITFA-A) procedure was used for TJPs (Fig. 2). In the follow-up all of the patients underwent MRI to exclude any tumor recurrence or to follow residual tumor size if exists. Demographic characteristics, presenting symptoms and signs, radiologic findings, operative techniques, complications and follow up data were all reviewed in detail.

### Histopathological analysis

All pathological specimens were analyzed under light microscopy. The characteristic arrangements of the chief cell nets (Zellballen) were examined. Antibodies against chromogranin, synaptophysin and S-100 protein were routinely used in immunohistochemical analysis.

### Statistical analysis

The statistical analysis was performed by using SPSS version 20.0 (Chicago, IL) statistical software package. The descriptive statistics were presented as mean ± standard deviation (SD). Continuous variables were tested by Kolmogorov–Smirnov test, histograms and P–P test for normality. Pearson Chi-Square test was used for comparing nominal variables. The numeric parameters were compared by Wilcoxon Signed Rank Test. In all the tests *p* < 0.05 was considered to be significant statistically.

## Results

There were 34 patients operated for TBPs in our clinic in the last 15 years. Of these 34 patients, 18 (52.9%) cases were operated for TMP and the remaining 16 (47.1%) cases were operated for TJP. The mean age was 50.3 ± 11.7 (range 25–71 years). The female/male ratio was 1.8:1 (22 females and 12 males). In particular female/male ratio were 2.6:1 and 1.2:1 for TMPs and TJPs, respectively. The tumor was located on right side in 16 (47.1%) cases whereas it was located on the left side in the remaining 18 (52.9%) patients. Multiple paragangliomas were observed in two cases with TMP (5.8%). One of the patients had TMP and glomus caroticum (GC) on the left and GC on the right side. She was examined for the presence of genetic mutation and succinate dehydrogenase (SDH) mutation was observed. The first-degree relatives were also examined for the presence of any glomus tumors with clinic examination and imaging. Familial penetrance was not observed. The second case had TMP on right side and GC on left side. Presenting symptoms are shown in Table 1. The most common presenting symptoms were tinnitus and hearing loss for both TMPs and TJPs. Facial paralysis, dysphagia and hoarseness symptoms were only encountered in TJPs. Catecholamine levels were studied in three patients with TJP and one case of TMP with multiple paraganglioma. We did not find increased catecholamine levels in plasma or urine samples in any of them.

**Table 1:** Presenting symptoms for temporal bone paragangliomas.

	TMP	TJP	Total (%)
Hearing loss	12	10	22 (64.7)
Tinnitus	16	10	26 (76.4)
Aural fullness	–	1	1 (2.9)
Vertigo	3	1	4 (11.76)
Otorrhagia	1	1	2 (5.8)
Otorrhea	1	1	2 (5.8)
Facial paralysis	–	3	3 (8.8)
Dysphagia	–	1	1 (2.9)
Hoarseness	–	1	1 (2.9)

### Audiological findings

The mean hearing thresholds for bone and air conduction with pure tune audiometry were 19.3 ± 11.8 and 39.5 ± 21.3 dB respectively. Therefore the mean gap between air-bone conduction in the affected side was 19.4 ± 14.5 dB (range 0–43). In particular the mean air-bone conduction gaps for TMPs and TJPs were 16.6 ± 14.8 (range 0–40) and 25.5 ± 12.7 (range 0–43), respectively (*p* = 0.219).

### Tumor characteristics

Tumor involvement was classified to be Fisch type A for 9 (50%) cases and, Fisch type B (50%) in the remaining 9 cases of TMPs. TJP tumors were classified to be C1, C2 and Di1 for 11, 4 and 1 case respectively as shown in Table 2. The mean maximum tumor size in axial plane for TMPs and TJPs was 11.2 ± 5.2 mm (range 5–23) and 30.2 ± 10.0 mm (range 15–70), respectively (*p* = 0.001). DSA was performed totally in 15 cases (46.9%). In particular it was performed for 9 (50.0%) cases with TMP and 7 (50.0%) cases with TJP. Afferent vessels identified with DSA are listed in Table 3. In some cases there were more than one afferent vessel. However in four cases with TMP there was not any afferent vessel identified with DSA. In one case with TMP the tumor located in tympanic cavity was not observed with DSA. The most common afferent arteries for TMPs and TJPs were found to be posterior auricular artery and occipital artery, respectively. Embolization was performed totally in 8 (23.5%) cases.

**Table 2:** Tumor involvement according to Fisch classification.

	TMP (*n*)	TJP (*n*)	Total (*n*)
Type A	9	–	9
Type B	9	–	9
Type C1	–	11	11
Type C2	–	4	4
Type Di1	–	1	1
Total (*n*)	18	16	34

**Table 3:** Afferent vessels identified with DSA.

	TMP (*n*)	TJP (*n*)	Total (*n*)
Ascending pharyngeal artery	2	3	5
Occipital artery	2	6	8
Posterior auricular artery	4	4	8
Middle meningeal artery	–	1	1
Total (*n*)	8	14	22

In any case, the tumor may have more than one afferent vessel.

### Surgical results

Gross total excision of tumor was achieved in nearly all cases (17/18 94.4%) for TMPs. 4 cases were operated via canal wall down tympanoplasty. In the remaining 8 cases 2 were operated via transcanally and 6 cases were operated with tympanoplasty and mastoidectomy plus posterior tympanatomy procedure. However gross total excision was achieved in only eight cases (8/16 50.0%) for TJPs (*p* = 0.003). In particular total excision was achieved in 5 (80%) cases among 6 cases with TJP who had undergone preoperative embolization. However total excision was achieved in 3 (30%) cases among 10 patients who had not undergone preoperative embolization (*p* = 0.039) (Table 4). Of these 8 cases with subtotal tumor resection, 5 cases (62.5) have been given stereotactic radiotherapy because of tumor growth in the follow up. All of the remaining cases are under control without an increase in tumor size on control MRI. For all the patients the mean follow-up period was 25.8 (range 4–108) months. In particular the mean follow-up was 40.1 (range: 10–108) and 10.2 (range: 1–26) months for TJPs and TMPs, respectively.

**Table 4:** Detailed information of patients with TJP.

*N*	Gender	Age	PE	Presentation/complications/management	Surgery	Tumor recurrence/growth	SRT	Follow-up/months
1	M	60	Yes	Presented with HB grade 5 facial palsy	Total	No	–	10
				Facial nerve sacrificed/CN 7–12 anastomosis-golden implantation to upper eyelid				
2	F	48	Yes	None	Subtotal	No	No	13
3	F	51	Yes	Revision case	Total	No	–	15
				Presented with HB grade 4 facial palsy				
				Hearing loss, otalgia CSF leakage/acetazolamid				
4	F	55	Yes	Recovered with HB grade 2 in postoperative one year	Total	No	–	18
5	M	49	Yes	None	Total	No	–	24
6	F	58	No	None	Subtotal	No	No	48
7	F	46	No	None	Subtotal	No	No	52
8	M	34	No	CN 10,12 sacrificed/thyroplasty	Total			18
9	M	49	No	HB grade 4 facial palsy/golden implantation to upper eyelid one year later: HB grade 2	Subtotal	Yes	Yes	53
				CSF leakage/lumbar drainage				
				Total SNHL				
10	F	29	No	None	Subtotal	Yes	Yes	35
11	M	53	No	Revision case	Subtotal	Yes	Yes	60
				Facial palsy (HB grade 3)				
				Total SNHL				
12	M	38	No	Facial palsy (Total)/CN 7–12 anastomosis-golden implantation to upper eyelid.-	Total	No	–	108
				Cholesteatoma				
13	F	61	No	Facial palsy (HB grade 4) 1 year later: HB grade 2	Total	No	–	66
14	F	71	Yes	Presented with facial palsy (unknown grade)	Total	No	–	12
				None				
15	M	25	No	Revision case	Subtotal	Yes	Yes	15
				Operated for chronic otitis media previously, previous biopsy result: hemangioma				
				External carotid artery sacrificed				
16	F	40	No	None	Subtotal	Yes	Yes	96

PE, preoperative embolization; SRT, stereotactic radiotherapy.

### Complications

The most common operative complication was facial nerve palsy during temporal paraganglioma procedures in our clinic. Five patients with TJP (31.2%) experienced facial palsy. Three patients with HB grade 4 after the operation were managed with gold weight implantation to upper eyelid. In the first year they have an acceptable recovery as HB grade 2 facial palsy. The facial nerve was sacrificed in two cases (one of them has already presented with HB grade 5 facial palsy). Hypoglossal-facial nerve end-to-end anastomosis has been performed in these patients. The external carotid artery was sacrificed in one case of TJP. Otorrhea and tympanic membrane perforation was observed in 3 patients. One of them (TJP case) developed iatrogenic cholesteatoma. Total sensorineural hearing loss was documented in three cases. One patient exhibited a co-existence of vestibular Schwannoma (VS) and TMP. Initially she underwent a revision surgery for total excision of TMP in our clinic. Soon after VS on the left side was excised totally via retro-sigmoid approach. Eventually she has ended up with bilateral total sensorineural hearing loss (SNHL); hearing restoration was achieved after cochlear implantation to the right side. The operation was aborted after subtotal tumor removal due to severe bleeding in one case with TJP. Cerebrospinal fluid leak occurred in two patients with TJP. One of them was treated with lumbar drainage and the other was given oral acetazolamide for four days. In one case with TMP, CSF leak occurred after tumor removal from internal acoustic channel. The small aperture was obliterated with a muscle graft during the operation. There was only one case with postoperative lower cranial nerve palsy (CN 10, CN 12) in one case of TJPs. He was managed with thyroplasty. Operative details and complications for TJP cases are given in detail in Table 4. In addition we did not observe any mortality owing to treatment failure or operative complications of TBPs in our patients.

## Discussion

Head and neck PGs are commonly accepted as slowly growing locally invasive benign tumors arising from parasympathetic fibers located along neurovascular structures.^4^ Only metastasis to cervical lymph nodes or distant organ such as lung or bone is considered as malignancy criterion, which accounts for less than 5% of cases.^4^ Carotid bifurcation is the most common site for PGs in head and neck region.^3^, ^4^ PGs of the temporal bone commonly originate from parasympathetic fibers located along adventitia of jugular bulb and tympanic plexus, so called as TJP and TMP.^15^ Middle age occurrence and female predominance is reported TBPs in the literature.^16^, ^17^ Likewise in our study the female/male ratio and the mean age were found to be 1.8:1 and 50.26 respectively. In addition female predilection was more apparent for TMPs (2.6:1) compared to TJPs (1.2:1) as compatible with the literature findings.^16^, ^17^ The most common presenting symptoms for TBPs are tinnitus and hearing loss.^6^, ^18^, ^19^ However TJPs may also present with symptoms indicating cranial nerve involvement.^4^, ^19^ In our series the most common presenting symptom was also tinnitus (76.4%) and hearing loss (64.7%) for TBPs. Four cases with TJP presented with symptoms of facial paralysis, dysphagia and hoarseness, indicating cranial nerve involvement. Chronic bloody otorrhea may be the presenting symptom in some cases.^6^ Likewise chronic otorrhea was the major presenting symptom in 2 cases (5.8%) in this study. Increased endocrine activity is very rare and the incidence is reported to be between 1% and 8% for TBPs.^20^ Therefore examination of urine and catecholamine levels is only advised in the presence of clinical symptoms like paroxysmal hypertension and flushing. We did not also observe increased catecholamine levels in blood and urine analysis of four patients who have been examined because of clinical suspicion. Mutations in SDH subunits are well established for familial occurrence and multiple paragangliomas.^10^ In our series SDH subunit mutation was only studied and shown in one case with sporadic multiple paraganglioma (bilateral glomus caroticum, unilateral TMP).

There are many classification systems suggested for staging head and neck paragangliomas. Fisch, Glasscock-Jackson and Shamblin classifications are those most commonly used.^13^, ^14^, ^21^, ^22^ In Fisch classification TMP and TJP are classified together as TBPs.^13^, ^14^ Therefore we also used Fisch classification system. In the present study tumor involvement was classified to be Fisch type A for 9 (50%) cases and, Fisch type B (50%) in the remaining 9 cases for TMPs. TJPs were classified to be C1, C2 and Di1 for 11, 4 and 1 case respectively. As expected the case with Di1 stage resulted in CSF leakage. The mean maximum tumor size in axial plane was significantly larger for TJP compared to TMP cases. It was calculated to be 11.2 ± 5.2 mm (range 5–23) and 30.2 ± 10.0 mm (range 15–70) for TMPs and TJPs, respectively (*p* = 0.001). Carlson et al. reported their series of 16 patients with TJPs. In their study the median maximum linear dimension for TJPs in the axial plane was found to be 2.0 cm (range, 1.4–3.9 cm).^18^

Carlson et al. also presented their experience on TMPs with 115 patients over 4 decades.^6^ Gross total removal was achieved in 108 (93.9%) patients. In the 7 patients (6.1%) with subtotal resection, the tumor was adherent to the petrous carotid artery, facial nerve, stapes footplate or round window. No recurrence was observed at a mean follow up of 30.4 months. Preoperative mean air conduction and air-bone gap with pure tune audiometry were found to be 40.6 and 17.1 respectively.^6^ In the current report there was one patient (5.5%) with subtotal excision among 18 patients with TMPs. The tumor was adherent to facial nerve in this case. The mean preoperative air-bone conduction gaps for TMPs and TJPs were 16.6 ± 14.8 (range 0–40) and 31.3 ± 6.2 (range 25–43), respectively (*p* = 0.219).

The main treatment modalities for TBPs are surgery and radiotherapy. Total tumor resection without major complications like cranial nerve deficit is the main goal of the operation. Canal wall up or down tympnomastoidectomy with or without facial recess approach is commonly adequate for the removal of TMPs.^6^ However IFTA-A is frequently needed for the total removal of TJPs, especially in case of intracranial extension. As defined by Fisch in 1982 it includes a cervical extension of a post-auicular incision to maintain best neurovascular control by exposing jugular bulb, jugular vein and carotid artery following mastoidectomy, facial rerouting, cervical incision and blind closure of the ear canal.^13^, ^14^ Salt and pepper appearance meaning areas of high and low intensity on MRI is typical for PGs.^23^ Nevertheless DSA may be performed to confirm the diagnosis in case of suspicion.^23^ In surgical candidates DSA may be performed just prior to the operation for selective embolization. Ascending pharyngeal artery and its branches are known to be the main feeding vessel for TBPs.^23^ However in the present study in the order of incidence occipital artery, posterior auricular artery and ascending pharyngeal artery were found to be most common afferent vessels. In four cases with TMPs there was not any afferent vessel identified with DSA. Preoperative embolization is not advised for Fisch class A tumors while it is strongly recommended for Fisch class C and D tumors. The main objective of preoperative embolization is to reduce vascularity and thus bleeding during surgery. In the retrospective study of Murphy et al., intraoperative bleeding, operation time and hospital stay for TJPs, were shown to be significantly reduced after preoperative embolization.^24^ However preoperative embolization for TBPs may also cause permanent cranial nerve palsy.^25^

In our series of 18 cases with TMPs and 16 cases with TJPs gross total tumor resection was achieved in 17 (94.4%) and 8 (50.0%) cases, respectively. Our overall subtotal excision rate (50%) was found to be very high compared to Gruppo Otologico experience for TJPs. Sanna et al. (Gruppo Otologico) have achieved 90.7% gross total excision in 55 patients over a period of 15 years. Their overall surgical control rate was 83% when residual tumors and recurrences were taken into account.^26^ We changed our management protocol for TJPs in recent years. All of the cases operated for TJPs (5 cases) with class C tumors in the last five years received preoperative selective embolization. Gross total excision was achieved in 5 (80%) cases among 6 cases with TJPs who have undergone preoperative embolization. However gross total excision was achieved in 3 (30%) cases among 10 patients who did not receive preoperative embolization (*p* < 0.05). From 8 cases with residual TJPs, five were subjected to SRT because of residual tumor growth. The remaining 3 cases were followed with serial MRI without evidence of tumor growth.

Nevertheless in spite of good surgical exposure with IFTA-A and preoperative embolization, gross total tumor removal cannot be achieved without possible major complications like lower cranial nerve deficit. Wanna et al. evaluated tumor growth after subtotal resection of large TJPs in 12 cases with functioning lower cranial nerves. In the mean 44.6 months follow-up, no tumor growth was observed in the residual tumor in 8 cases (66.6%). From four cases with the evidence of tumor growth in the residual tissue, two of them were given SRT.^27^ Karaman et al. achieved gross total resection with IFTA-A after preoperative embolization in 9 of 11 cases with TJPs. SRT was given for residual tumors.^19^ Therefore subtotal resection is also an advisable surgical option in TJPs.

Among operative complications of TBPs, facial nerve palsy is one of the most commonly encountered.^11^, ^19^ In our series 5 patients (31.2%) with TJPs experienced facial palsy owing to operation. All of them except two improved with HB grade 2 and 3 in the follow up. Hypoglossal-facial nerve end-to-end anastomosis was needed in two cases owing to facial nerve sacrifice (one of them has already presented with HB grade 5 facial palsy before the operation). One of the TJP cases who initially presented with HB grade 4 facial palsy recovered with HB grade 2 following the operation. Chronic otorrhea may be a primary presenting symptom or due to a complication of surgical procedure in TBPs.^19^ In the current study three patients experienced otorrhea and tympanic membrane perforation after the procedure. In one of our patients, who underwent revision surgery for chronic otorrhea developing after the primary operation for TJP, the final histopathologic examination revealed an iatrogenic cholesteatoma probably due to incorrect cul-de sac technique. To our knowledge it is the third case of acquired cholesteatoma developing after TJP operation reported in the English literature.^28^, ^29^ There was also a co-existence of VS and TMP in our series. After the treatment of TMP and VS, she has ended up with bilateral total SNHL and hearing restoration was achieved after cochlear implantation to the right side. To our knowledge this is the third case regarding co-existence of VS and TMP and it is the first case who underwent cochlear implantation for simultaneous occurrence of VS and TMP in the opposite locations in the international literature.^30^

As mentioned above the only curative treatment is total excision for TBPs. However, in case of large tumors and advanced age, radiotheraphy or observation without further intervention must be considered. Lee et al. presented their experience with gamma knife radiosurgery for TJPs and TMPs. The median tumor volume reduction was found to be 34.0% in a median 40.3 months follow-up time. There was only one patient with facial nerve paralysis and hearing impairment after treatment. The tumor control rate was reported to be 100%.^31^ Carlson et al. reported their experience with observation for TJPs in 16 patients. The major indication for observation was patient preference and advanced age. In serial radiologic examination among 12 patients 5 patients (42%) demonstrated radiologic growth, while 7 (58%) remained stable.^18^ Van der Mey et al. compared results of surgery, radiation therapy and observation protocols. In their 36 years follow-up series of 105 patients, no difference revealed regarding survival rates between these groups.^32^

## Conclusion

In conclusion, based on our findings and literature review, total surgical excision alone or in conjunction with preoperative embolization and total surgical excision with preoperative embolization, are the main treatment modalities for TMPs and TJPs, respectively. However radiotherapy and observation modalities are also considered in case of large tumors, recurrent cases, advanced age and patient preference. In addition subtotal resection must be considered rather than sacrificing major neurovascular structures in case of preoperative functioning cranial nerves and large tumors. Besides tinnitus and hearing loss, otorrhea may also be either a presenting symptom or a postoperative finding pointing to tumor recurrence or a complication like cholesteatoma for TBPs. Additionally to our knowledge we have reported the first case who has received cochlear implantation for simultaneous occurrence of VS and TMP in the opposite locations.

## Conflicts of interest

The authors declare no conflicts of interest.
